# Long Segment Coarctation of the Abdominal Aorta in a 12-Year-Old Patient: A Case Report

**DOI:** 10.7759/cureus.100054

**Published:** 2025-12-25

**Authors:** Debashish Nayak, Samarjit Bisoyi

**Affiliations:** 1 Cardiovascular and Thoracic Surgery, Apollo Hospitals Bhubaneswar, Bhubaneswar, IND; 2 Anesthesia, Apollo Hospitals Bhubaneswar, Bhubaneswar, IND

**Keywords:** congenital vascular anomaly, dacron graft, long segment coarctation, middle aortic syndrome, pediatric hypertension

## Abstract

Coarctation (CoA) of the abdominal aorta, better classified as midaortic syndrome (MAS), represents a rare and complex vascular anomaly that results in narrowing of the descending distal thoracic and/or abdominal aorta. Clinical presentation in pediatric patients is complex and sometimes undetected; consequently, prognosis is grim, especially in preterm infants. Most common indications are hypertension, which mostly remains unresolved with high doses and/or combinations of antihypertensive medications, claudication of lower limbs and/or feeble or absent femoral pulses. Surgery appears to be the only preferred choice of treatment in pediatric patients for lifelong disease management. In this case presentation, a 12-year-old male child exhibited severe headache and myalgia over the past six months. Physical examination revealed resting blood pressure of 146/80 mmHg despite being on three antihypertensive medications. A Doppler test and CT angiogram confirmed the presence of long-segment coarctation of the distal thoracic and proximal abdominal aorta. Other reports, such as blood tests, ECG and 2D echocardiogram, were normal. The patient thereafter underwent successful thoraco-abdominal aorto-aortic bypass employing a Dacron tube graft (14 mm x 60 cm), which resulted in an immediate drop in the brachio-femoral gradient (radial-160/85 mmHg, femoral-120/80 mmHg, mean gradient 16.6 mmHg). The patient was discharged on postoperative day (POD) 12, and the antihypertensive medications were readjusted. In subsequent follow-up periods for up to two years, all antihypertensive medications were stopped. At five years POD, the blood pressure rebounded to 170/94 mmHg. The CT angiograms post-surgery at one-month and five-year POD were normal. The patient did not report any adverse event (AE) and remained asymptomatic for five years.

## Introduction

Coarctation (CoA) of aorta is a congenital heart disease commonly observed in the thoracic cavity, and it reportedly accounts for 5-8% of all reported congenital heart defects [1]. CoA of the abdominal aorta has also been reported; however, it is a rare and diagnostically challenging vascular anomaly which was reported for the first time by Quain in 1947 [2]. The World Health Organization (WHO) does not have specific statistics on the prevalence of CoA of the abdominal aorta; nonetheless, reports from other studies reveal an incidence of 2% [1] or 1-3% [3] of all aortic CoAs. It is mostly prevalent in children and young adults, with an average age of diagnosis being 20.7 years [1]. The clinical picture of such patients presents with uncontrolled hypertension, abdominal claudication or lower limb claudication, or visceral ischemia.

Its etiopathogenesis is mostly controversial and varies depending on whether the condition is congenital or acquired [1]. In the congenital form, there is an error during embryonic development when the paired dorsal aortae fuse to form the single midline abdominal aorta [4]. Hypoplasia of the abdominal aorta results from irregular fusion or localized obliteration of the lumen in one of the paired aortae. Acquired etiopathogenesis is often reflected later in life, owing to underlying diseases, including Takayasu's arteritis, neurofibromatosis, fibromuscular dysplasia, retroperitoneal fibrosis or mucopolysaccharidosis [5-8].

Diagnosis of CoA of the abdominal aorta spans from simple physical examinations such as blood pressure and pulse checks to complex imaging studies such as echocardiogram, CT angiography and magnetic resonance angiography. Other tests include electrocardiogram, cardiac catheterization and exercise stress test. CT angiography is the preferred choice as it provides excellent and detailed anatomical information [9]. CoA of the abdominal aorta can be confused with Takayasu's arteritis owing to similar clinical symptoms and it is important to distinguish between the two. Takayasu's arteritis is an inflammatory condition affecting large blood vessels, while CoA is a congenital narrowing of the aorta [2]. Management strategies aim to eliminate the narrowed segment of aorta and restore the normal blood flow. Depending on factors such as the patient's age and severity of the CoA-associated cardiac or other anomalies, treatment is usually achieved through surgical or transcatheter procedures. In critical patients, particularly neonates, cardiorespiratory support and prostaglandin E1 infusion have shown to improve blood flow through the ductus arteriosus. Definitive treatment options include surgical procedures and transcatheter procedures. Procedures such as aorto-aortic bypass, thoracoabdominal bypass, and patch aortoplasty have shown favorable outcomes in the treatment of CoA of the abdominal aorta [5,10,11]. The present case describes a 12-year-old male patient with long-segment distal thoracic and proximal abdominal aortic CoA, who underwent successful thoraco-abdominal aorto-aortic bypass using a Dacron conduit, with excellent early and late outcomes.

## Case presentation

A 12-year-old male patient presented with severe headache and musculoskeletal pain over the past six months. On examination, his vital parameters at baseline were: heart rate 94/min (reference range: 70-110 min), respiratory rate 21/min (reference range: 12-35/min), SpO2 100% (reference range: 95-100%) on room air. Blood pressure (BP) in the left arm resting supine was 140/86mm Hg, despite being on three anti-hypertensive drugs.

Further physical examination revealed that bilateral lower limb pulsations were impalpable. No radiofemoral delay was observed, and the patient did not present with any other symptoms. Further investigation, employing an abdominal ultrasound with Doppler test, to assess the cause of hypertension, led to the identification of small-diameter stenosis (82%) of the abdominal aorta with gradient turbulence across the segment.

**Figure 1:**
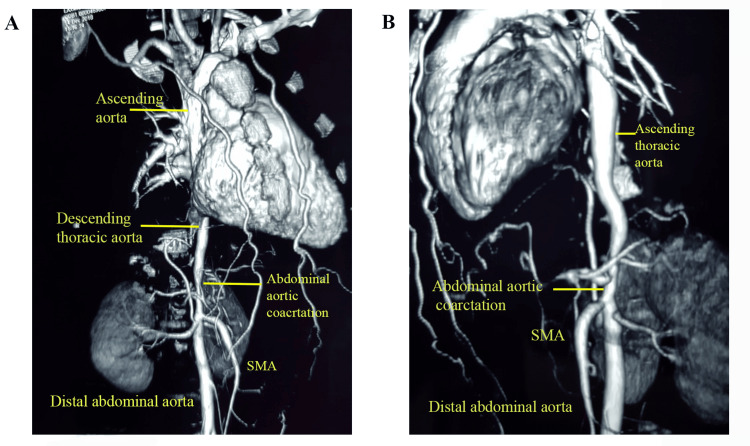
CT aortogram showing coarctation of the abdominal aorta (A) Apical view showing the coarctation segment (8 mm diameter) and distal abdominal aorta (16 mm diameter); (B) Lateral view showing the descending thoracic aorta and the SMA (superior mesenteric artery).

CT angiogram indicated long-segment CoA of the distal thoracic and proximal abdominal aorta. The ascending aorta and the descending thoracic aorta appeared normal, measuring 22 mm and 15 mm in diameter, respectively. The CoA segment of the abdominal aorta was 8 mm, and the distal abdominal aorta was 16 mm in diameter (Figures 1A, 1B).

The major abdominal arteries, such as the renal arteries and superior and inferior mesenteric arteries, appeared normal. However, an insignificant ostial block of the superior mesenteric artery (SMA) of ~30% was detected. The blood tests, 2D echo and ECG were normal, and no other congenital anomalies were detected during physical examination or from the tests that were performed.

After completion of all the tests, the patient immediately underwent successful thoraco-abdominal aorto-aortic bypass grafting. During the surgery, he was placed supine with the left upper limb abducted. A long left thoraco-abdominal incision was made. Intraoperative invasive BP monitoring revealed elevated radial pressure at 170/90 mmHg (mean arterial pressure or MAP of 116.6 mm Hg), with low femoral pressure of 70/30 mmHg (MAP 43.3 mmHg). A peak systolic gradient of 100 mmHg and a mean gradient 73.2 mmHg confirmed a significant pressure gradient.

**Figure 2:**
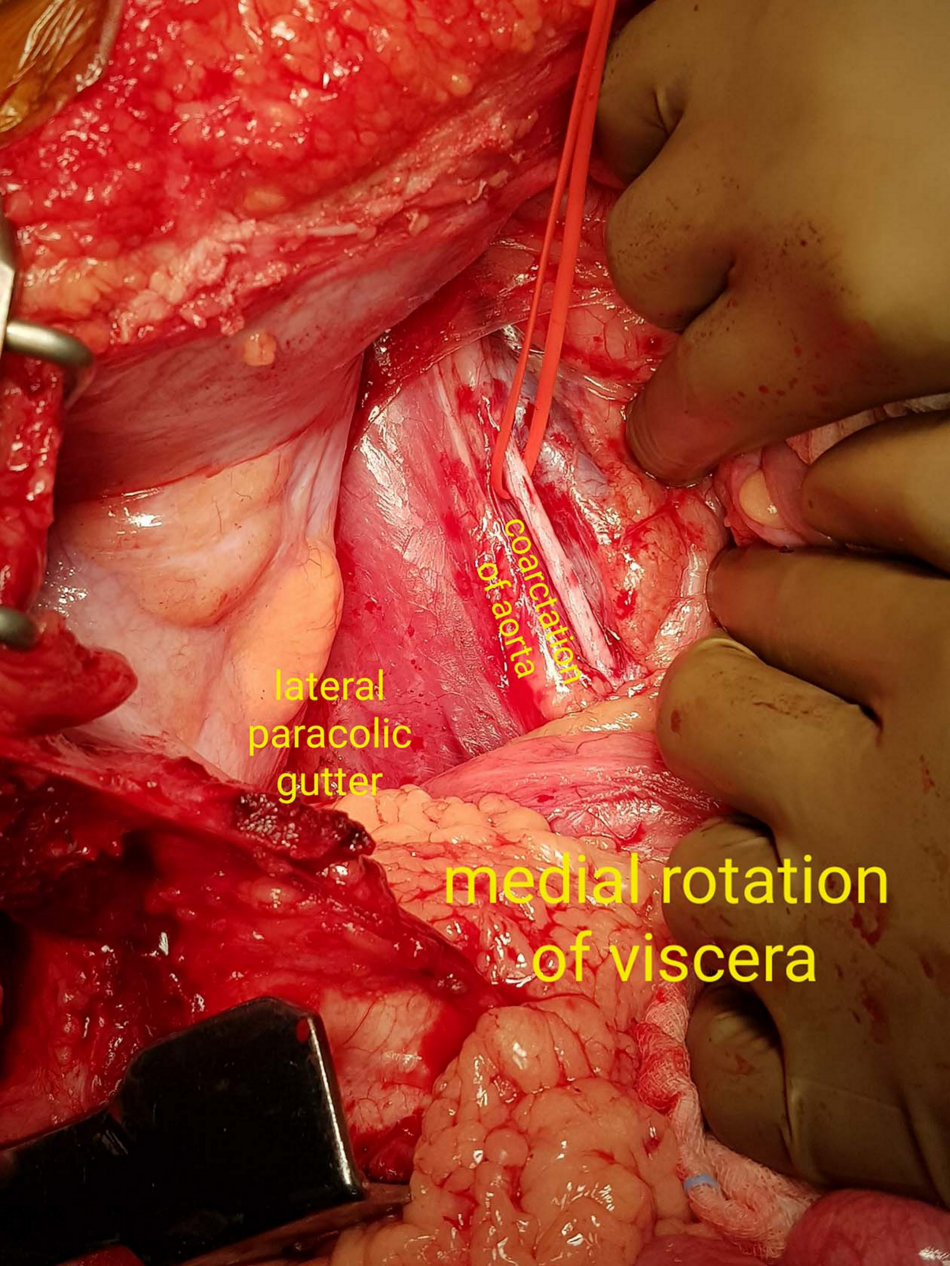
Operative image showing medial visceral rotation (Mattox manouvre) and exposure of the abdominal aorta (in the red silastic loupe)

The left thorax was entered from fifth intercostal space and the abdomen opened midline. Then a left medial visceral rotation was performed wherein an incision was made in the peritoneal reflection in the left para-colic gutter and the viscera was rotated medially and to the right, in order to completely expose the retro-peritoneal abdominal aorta (Figure 2).

**Figure 3:**
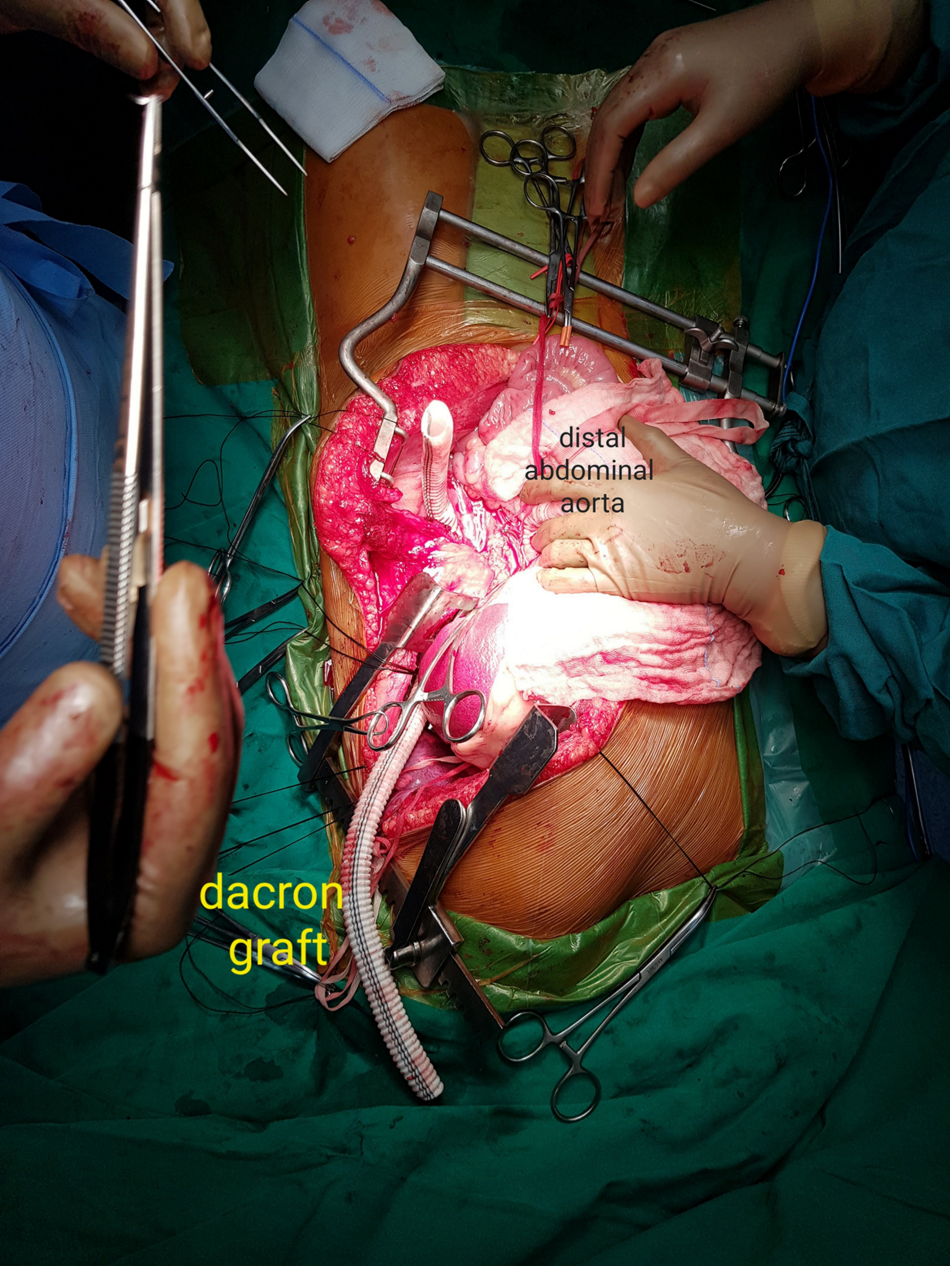
Operative image showing the dacron graft being anastomosed proximally and distally

The diaphragm was then incised circumferentially and the entire thoracic aorta was similarly exposed. The thoracic aorta was found to be normal in its proximal part but stenosing in its distal portion, just 3 cm proximal to its passage into the abdomen. This part of the aorta was found to be diffusely thin, measuring approximately 8-9 mm in diameter. A commercially available Dacron tube graft (Getinge AB, Goteburg, Sweden) of 14mm x 60 cm was placed in a curved ‘C’ configuration and anastomosed proximally to thoracic aorta and inferiorly to abdominal aorta just above bifurcation, passing through a defect created in the postero-lateral part of the diaphragm (Figure 3).

**Figure 4:**
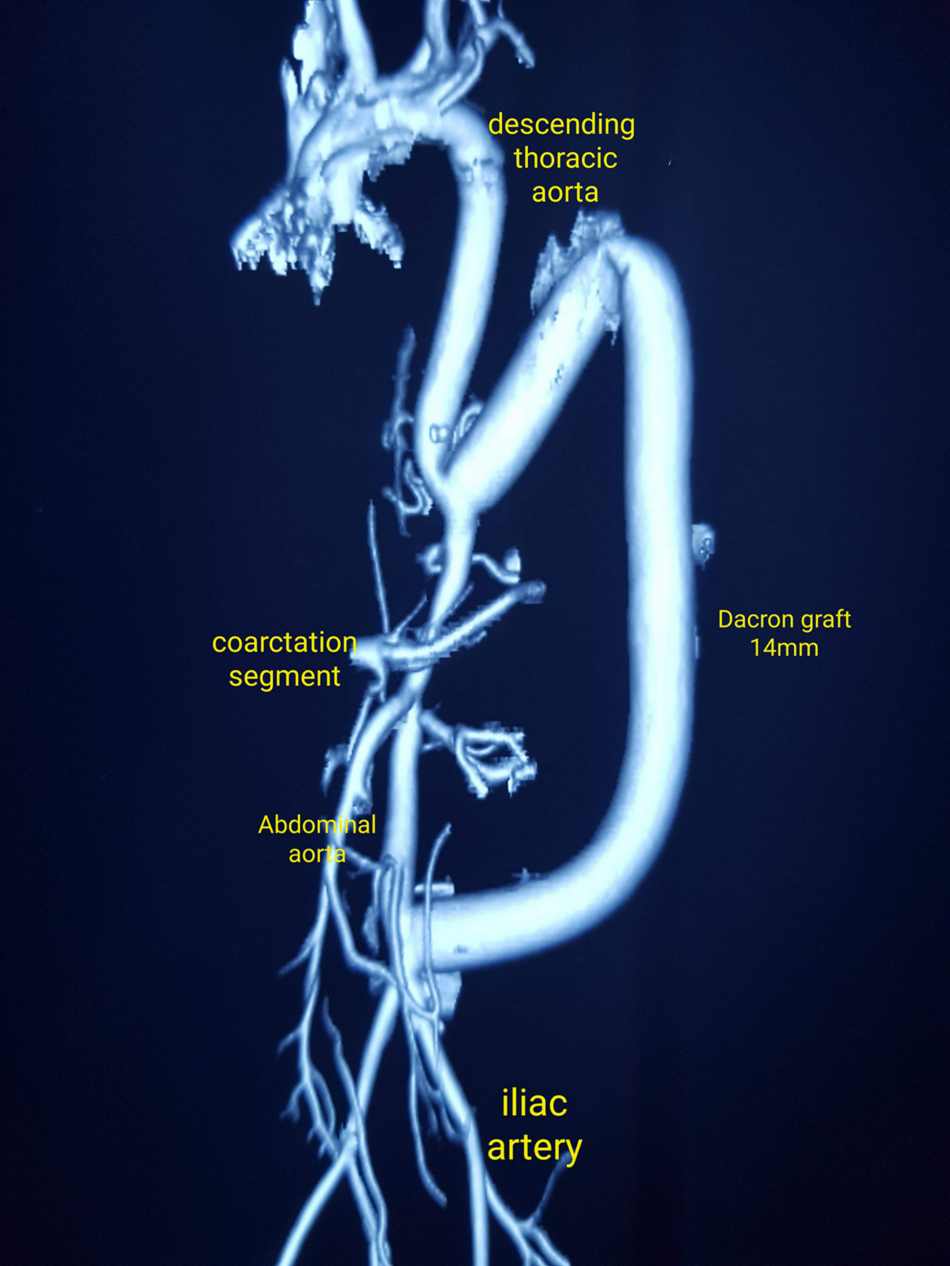
CT aortogram at one month post-surgery showing the Dacron graft in a C-shaped loop

This resulted in an immediate drop in the brachio-femoral gradient (radial-160/85 mmHg, femoral-120/80 mmHg, mean gradient 16.6 mmHg) and the distal pulsations in both the lower limbs were then palpable. The peritoneum was resutured, the diaphragm was repaired, and the chest and abdomen were closed in a routine fashion. Post-op recovery was smooth and uneventful. The gradient started to reduce gradually from post-operative day (POD) two onwards. At discharge, on POD 12, the patient’s pressure recordings were: left brachial, 118/65 mmHg and left femoral, 118/67 mmHg. Subsequently, all antihypertensive medications were stopped and a post-op CT angiogram done at one month showed excellent results (Figure 4).

Subsequent follow-up period was uneventful, and the patient remained asymptomatic until the two-year follow-up. The wounds were completely healed, the pedal pulses were well-felt, and the BP returned to normal without pharmacological intervention.

**Figure 5:**
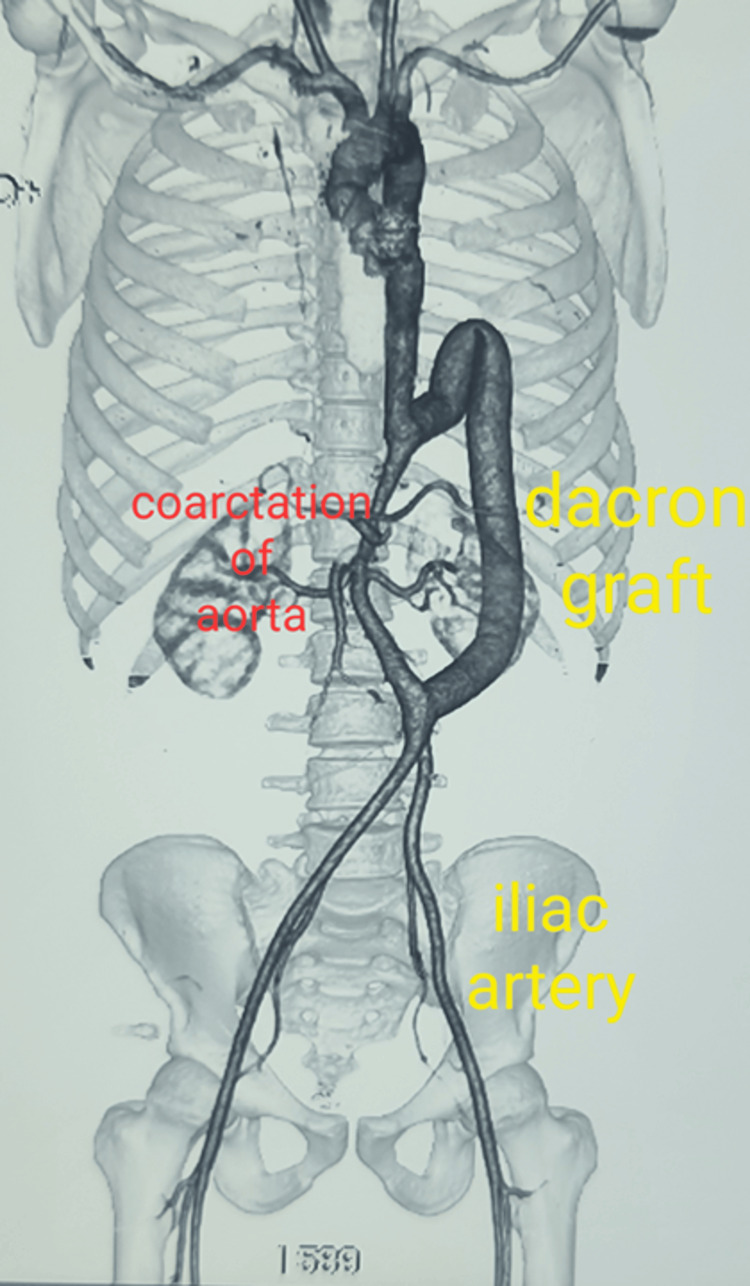
CT aortogram at the five-year follow-up showing good flow in the Dacron graft and good distal flow

At the five-year follow-up, his BP shot up to 170/94 mmHg, left brachial and left femoral pressure was 170/100 mmHg, with a heart rate of 102/min and SpO2 of 100% on room air. On reversal of hypertension, the patient was prescribed amlodipine 10mg once daily. The BP was controlled and was 150/84 mmHg one month since the antihypertensive medication was started. Figure 5 shows the normal CT aortogram at the five-year follow-up.

## Discussion

The term 'middle aortic syndrome' (MAS) was coined by Sen and colleagues in 1963 [12] to represent a rare group of patients, atypically presenting with localized or diffused stenosis of the thoracoabdominal aorta, which is congenital, genetic or idiopathic in nature. This paper presents a rare congenital case of MAS, with a severe stenosis of the abdominal aorta (8 mm in diameter), which was successfully operated by a Dacron tube graft. This resolved the blood pressure and normalized the brachio-femoral gradient post-surgery to a point where the patient remained asymptomatic for two years, and all medications were stopped. Five years after the surgery, the hypertension resurfaced, and the patient's BP shot up to 170/90mm Hg, and he was, therefore, put on amlodipine 10 mg once daily. The CT angiogram after five years was normal, and the patient remained asymptomatic. This is possibly a rare case scenario wherein a patient with MAS has remained asymptomatic with minimal drug administration post-surgery, with no adverse event (AE) reported either during the peri-operative or post-operative period. 

Although MAS is a rare syndrome, more reports of MAS are surfacing in the recent years owing the advanced and sophisticated diagnostics associated with the state-of-the-art therapeutic interventions. Despite this feat, reports on outcomes of the disease still remains sparse. Reportedly, idiopathic cases are relatively more common compared to congenital and genetic disorders [13]. In a recent Indian retrospective study reported by Kota and colleagues with a study pool of 33 patients, only one patient reported no requirement of antihypertensive medications, 75% of them required at least one antihypertensive medication whereas in 28% of the patients, the hypertension remained uncontrolled. Since the data was collected over a period of three decades and although average follow-up period was mentioned as nine years, there was no clarity on the outcome of the patients on an individual level for a particular duration [14]. Most AEs reported post-surgery include non-flow-limiting dissection, restenosis after angioplasty, and in-stent stenosis [14]. In the present case, the patient remained asymptomatic without any medications for a period of up to two years; however, the hypertension resurfaced and was managed by a single medication. It remains worthwhile to mention here that the CT angiography was normal and no other AEs were observed.

Patients diagnosed with MAS during the early years present a severe form of the disease, and intervention during the initial years could be a cause for vascular complications. Often endovascular interventions fail to achieve controlled long-term BP values and hence surgical procedure is needed [13,15]. Aorto-aortic bypass remains a standard treatment modality, and reportedly more than half of the patients undergo this procedure, followed by patch aortoplasty, and primary aortic repair after aortic lengthening [16]. Some of the complications following surgical intervention include bleeding, clots, graft stenosis, thrombosis, and iatrogenic tears [16]. In this case, a standard intervention employing aorto-aortic bypass grafting was employed and the graft diameter was 14 mm, which was relatively wide keeping in mind adjustment with the growth of the child and also to accommodate good perfusion without the necessity of future corrective surgery [4].

Dacron grafts have proven to be more resilient than aortic thoracic endovascular aortic repair (TEVAR), with half of the patients requiring intervention within five years [17]. In another German study, a 15-year follow up of an infant who underwent an aorto-aortic bypass surgery at seven months, the length of the graft was adjusted to accommodate growth in adolescence and the patient did not report any aortic reoperation and/or lower limb malperfusion [18]. Go et al. reported long-term complications in two pediatric patients in USA who underwent aorto-aortic bypass grafting wherein one patient reportedly developed growth-related complications whereas the other patient developed a graft-enteric fistula [15]. In a meta-analysis on MAS involving 92 pediatric patients and 88 adults who were included from 121 studies, Cortenbatch and colleagues reported that adults, after endovascular treatment, presented with lesser degree of hypertension, whereas in juvenile patients, surgery decreased the probability of hypertension [19]. The authors however argued that endovascular therapy has evolved over the past two decades and therefore a certain degree of bias might be associated with their findings [19]. Another retrospective long-term follow-up study from China involving 41 adults (mean age; 37.5±3.4 years) aligns with the findings of Cortencatch and colleagues in that 92.3% and 79.1% adults undergoing endovascular treatment remained reintervention-free during a five- and 10-year follow-up, respectively as compared to open surgical treatment (87.7% and 71.7%) [20]. This is in line with the finding that pediatric patients responded better to surgical treatment with improved outcomes [19]. It is to be kept in mind that surgical reconstruction requires careful planning and sufficient technical expertise as aortic reconstruction appears to be an optimal choice of treatment in pediatric patients for better long-term outcomes [4].

Considering the complexity of the published data on MAS coupled with the paucity of the predictive factors that could influence the outcome, there are no clear guidelines on the diagnosis and management of the disease in children as most of the published data are derived from observational studies with low patient numbers. Moreover, various layers of heterogeneity such as etiology, medications, age groups, and vascular involvement further complicate the outcome scenario. In an already rare disease where patient numbers are scarce, another hurdle is that patients more often than not are lost to follow-up, possibly because their disease post-therapy becomes stable or they change their clinician or location. Nonetheless, lifelong follow-up in children is warranted to prevent future complications, owing to their young age. For better treatment outcomes, therefore, a multidisciplinary approach along with regular follow-up is pertinent.

## Conclusions

MAS is a rare vascular disorder that mainly impacts the abdominal aorta and is a serious cause for hypertension affecting children with a poor prognosis reported in preterm infants; however, adult cases with MAS have also been reported. Clinical manifestations mostly include hypertension, headache, claudication of the inferior limbs, high brachio-femoral gradient, abdominal bruit and/or feeble or absent femoral pulses. Pediatric patients mostly present with a congenital etiology, and without prompt intervention, their survival rate is poor.

Medical management of pediatric patients with antihypertensives is not an option, considering their young age. Therefore, surgery is the only preferred choice of treatment to ensure improved quality of life. Owing to its proven resilience, a Dacron graft was used in the aorto-aortic bypass, and the patient remained asymptomatic for two years. However, after five years, his hypertension reappeared, and he was medically managed with a single medication. The patient remained asymptomatic until five years post-surgery. Contrary to publications reporting AEs post surgical intervention, this report adds valuable insight into the surgical management of long-segment abdominal aortic hypoplasia in pediatric patients and underpins the need for early diagnosis to prevent hypertensive complications and end-organ damage. To the best of our knowledge, this is possibly the first report from India on successful aorto-aortic bypass surgery to manage MAS with promising outcomes even five years after surgery, with no reports of AEs and an improved quality of life.
